# Fine structure in the $\upalpha$ decay of $^{179}$Hg and $^{177}$Au

**DOI:** 10.1140/epja/s10050-025-01513-9

**Published:** 2025-03-07

**Authors:** A. Špaček, A. Herzáň, M. Sedlák, M. Venhart, F. A. Ali, A. N. Andreyev, K. Auranen, S. Bánovská, M. Balogh, R. J. Carroll, D. M. Cox, J. G. Cubiss, T. Davis, M. C. Drummond, J. L. Easton, T. Grahn, A. Gredley, P. T. Greenlees, J. Henderson, U. Jakobsson, D. T. Joss, R. Julin, S. Juutinen, G. Kantay, J. Kliman, J. Konki, E. A. Lawrie, M. Leino, V. Matoušek, A. K. Mistry, C. G. McPeake, D. O’Donnell, R. D. Page, J. Pakarinen, P. Papadakis, J. Partanen, P. Peura, P. Rahkila, A. Repko, P. Ruotsalainen, M. Sandzelius, J. Sarén, B. Saygi, D. Seweryniak, C. Scholey, J. Sorri, S. Stolze, A. Thornthwaite, I. S. Timchenko, J. Uusitalo, M. Veselský, S. Vielhauer, F. P. Wearing

**Affiliations:** 1 Heavy Ion Laboratory, University of Warsaw, Ludwika Pasteura 5A, 05-077 Warsaw, Poland; 2 Institute of Physics, Slovak Academy of Sciences, Bratislava, 84511 Slovakia; 3 Oliver Lodge Laboratory, University of Liverpool, Liverpool, L69 7ZE UK; 4 Department of Physics, College of Science Education, University of Sulaimani, Sulaimani, 334 Kurdistan Region Iraq; 5 School of Physics, Engineering and Technology, University of York, Heslington, YO10 5DD UK; 6 Advanced Science Research Center, Japan Atomic Energy Agency (JAEA), Tokai-mura, Naka-gun, Ibaraki 319-1195 Japan; 7 Accelerator Laboratory, Department of Physics, University of Jyväskylä, Jyväskylä, 40014 Finland; 8 INFN Laboratori Nazionali di Legnaro, 35020 Padova, Italy; 9 iThemba Laboratory for Accelerator Based Sciences, P.O. Box 722, 7129 Somerset West, South Africa; 10 Department of Physics and Astronomy, University of the Western Cape, Bellville, 7535 South Africa; 11 School of Computing, Engineering and Physical Sciences, University of the West of Scotland, PA1 2BE Paisley, UK; 12 Department of Physics, Faculty of Science, Ege University, 35100 Izmir, Turkey; 13 Department of Physics, Faculty of Science and Arts, Sakarya University, 54187 Sakarya, Turkey; 14 Physics Division, Argonne National Laboratory, Lemont, 60439 USA; 15 Institute of Experimental and Applied Physics, Czech Technical University, Prague, Czech Republic; 16 Present Address: Laboratory of Radio-Chemistry, Department of Chemistry, University of Helsinki, P.O. Box 55, 00014 Helsinki, Finland; 17 Present Address: CERN, 1211 Geneva 23, Switzerland; 18 Present Address: School of Computing, Engineering and Physical Sciences, University of the West of Scotland, Paisley, PA1 2BE UK; 19 Present Address: STFC Daresbury Laboratory, Daresbury, Warrington WA4 4AD UK; 20 Present Address: Helsinki Institute of Physics, P.O. Box 64, 00014 Helsinki, Finland; 21 Present Address: STUK - Radiation and Nuclear Safety Authority, P.O. BOX 14, 00811 Helsinki, Finland; 22 Present Address: Physics Division, Argonne National Laboratory, Argonne, IL 60439 USA

## Abstract

The $\upalpha$-decay fine structure of $^{179}$Hg and $^{177}$Au was studied by means of decay spectroscopy. Two experiments were performed at the Accelerator Laboratory of the University of Jyväskylä (JYFL), Finland, utilizing the recoil separator RITU and a digital data acquisition system. The heavy-ion induced fusion-evaporation reactions $^{82}_{36}$Kr + $^{100}_{44}$Ru and $^{88}_{38}$Kr + $^{92}_{42}$Mo were used to produce the $^{179}$Hg and $^{177}$Au nuclei, respectively. Studying the evaporation residues (ER, recoils)-$\alpha _1$-$\alpha _2$ correlations and $\upalpha$-$\gamma$ coincidences, a new $\upalpha$ decay with E$_\alpha$ = 6156(10) keV was observed from $^{179}$Hg. This decay populates the (9/2$^-$) excited state at an excitation energy of 131.3(5) keV in $^{175}$Pt. The internal conversion coefficient for the 131.3(5) keV transition de-exciting this state was measured for the first time. Regarding the $^{177}$Au nucleus, a new $\upalpha$ decay with E$_\alpha$ = 5998(9) keV was observed to populate the 156.1(6) keV excited state in $^{173}$Ir. Two de-excitation paths were observed from this excited state. Moreover, a new 215.7(13) keV transition was observed to depopulate the 424.4(13) keV excited state in $^{173}$Ir. Properties of the $^{179}$Hg and $^{177}$Au $\upalpha$ decays were examined in a framework of reduced widths and hindrance factors. For clarity and simplicity, the spin and parity assignments (e.g. $J^{\pi }$) are presented without brackets throughout the text.

## Introduction

The neutron-deficient nuclei in the vicinity of the closed proton shell at *Z* = 82 lie in a region with strong manifestations of shape coexistence. Studying the $\upalpha$ decays of these nuclei can provide useful information about underlying nuclear structure [1–3] and deformation of the studied nuclides [4–6]. The $\upalpha$-decay hindrance factor (HF) represents a ratio between the overlaps of the initial and final nuclear states for different decays [7]. This provides a link (a similarity or a difference) between the initial and final states.

In this work, results of the $\upalpha$-decay study of $^{179}$Hg and $^{177}$Au are presented. It is the first study in which the fine structure in the $\upalpha$ decay of $^{179}$Hg has been observed.

Prior to this work, several studies of the $^{179}$Hg $\upalpha$ decay were performed [8–14]. The $\upalpha$-particle energy for the ground state (g. s. ) to g. s. $\upalpha$ decay was reported to be 6288(5) keV with an $\upalpha$-decay branching ratio of 75(4) % [13] and a half-life of 1.05(3) s [15]. The hindrance factor for this decay was determined to be 1.4(2), supporting the $^{179}$Hg 7/2$^-$ g. s. $\rightarrow$ $^{175}$Pt 7/2$^-$ g. s. $\upalpha$-decay assignment. The Nilsson-configura-tions of 7/2[514] (2$f_{7/2}$) and 7/2[503] (1$h_{9/2}$) were suggested for the $^{179}$Hg ground state [14].

Regarding the decay product, $^{175}$Pt, altogether four $\upalpha$ decays are known at the time of our study. Three of these decay branches, with energies of $\upalpha$-particle equal to 6038(10), 5960(3) and 5831(10) keV, were studied in the past [10, 11, 16, 17]. Only recently, a 4$^{th}$ $\upalpha$-decay branch with E$_\alpha$ = 5819(4) keV was observed [18]. It feeds the 9/2$^-$ excited state at an excitation energy of 207.9(5) keV in $^{171}$Os. This excited state de-excites via emission of the 207.9(5) keV $\gamma$ ray to the 5/2$^-$ ground state or via the 130.8(4) keV $\gamma$ ray to the 7/2$^-$ excited state at 76.7(3) keV [18, 19]. In the same study [18], the 130.9(6) keV transition de-exciting a 9/2$^-$ excited state of $^{175}$Pt to its 7/2$^-$ ground state was also found. It was therefore desirable to search for an $\upalpha$ decay feeding the 9/2$^-$ excited state at 130.9(6) keV in $^{175}$Pt, and to study similarities between the $\upalpha$ decays of $^{175}$Pt and $^{179}$Hg, which could reveal a decay pattern in the above-described decay chain.

The $\upalpha$ decay of $^{177}$Au was studied in [17, 20–23]. Three $\upalpha$ decays were reported: E$_\alpha$ = 6156(5) keV: 1/2$^+$ g. s. $\rightarrow$ 1/2$^+$ g. s., E$_\alpha$ = 6125(5) keV: 11/2$^-$, 181.9(4) keV $\rightarrow$ 11/2$^-$, 213 (16) keV, and E$_\alpha$ = 5932(12) keV: 11/2$^-$, 181.9(4) keV $\rightarrow$ 9/2$^-$, 424.4(13) keV [23]. Partial $\upalpha$-decay branching ratios were reported to be 64(5) and 56(8) % with half-lives of 1.501(20) s and 1.193(13) s for the $\upalpha$ decays depopulating the g. s. and the 226(18) keV isomeric state in $^{177}$Au, respectively. The spin-parity of the $^{177}$Au g.s. has been unambiguously assigned as 1/2$^+$ [24]. The proposed Nilsson configuration is either 1/2$^+$[411] (2$d_{3/2}$) with oblate deformation, or 3/2$^+$[402] (2$d_{3/2}$) with prolate deformation and 11/2$^-$[505] (1$h_{11/2}$) configuration for the 11/2$^-$ isomeric state in $^{177}$Au [21].

In the present article, we report on the observation of a new $\upalpha$ decay of $^{179}$Hg, which allowed unambiguous determination of the multipolarity of the known 130.9(6) keV $\gamma$-ray transition in $^{175}$Pt. New $\gamma$-ray transitions attributed to the fine structure of the $^{177}$Au $\upalpha$ decay are also presented.

## Experimental details

The datasets discussed in this paper were collected in two separate experiments, both performed at the Accelerator Laboratory of the University of Jyväskylä (JYFL), Finland. The $^{179}$Hg isotope was produced through the $^{100}_{44}$Ru($^{82}_{36}$Kr, 3n) $^{179}_{80}$Hg fusion-evaporation reaction. The beam of $^{82}$Kr$^{15+}$ ions, accelerated to an energy of 352 MeV, was delivered by the K-130 cyclotron and impinged on a self-supporting $^{100}$Ru target with a thickness of 350 $\upmu$g/cm$^2$. The total irradiation time was 267 hours, with an average beam intensity of 5 *p*nA. The $^{177}$Au nuclei were produced via the $^{92}_{42}$Mo($^{88}_{38}$Sr, p2n)$^{177}_{79}$Au fusion-evaporation reaction. The $^{88}$Sr$^{10+}$ ion beam with an energy of 399 MeV bombarded a 600-$\upmu$ g/cm$^{2}$ thick $^{92}_{42}$Mo target (enrichment 98 %). The average beam intensity was 2 *p*nA and the beam-on-target time was 226 h. In both experiments, ER were separated from the primary ion beam and other unwanted particles by the gas-filled separator RITU [25, 26], and focused to the focal-plane GREAT spectrometer [27] for further analysis. In the GREAT spectrometer, the recoils first passed through a multi-wire proportional counter (MWPC) and were implanted into one of the two adjacent 300-$\upmu$ m thick double-sided silicon strip detectors (DSSD). Each DSSD had a size of 60 $\times$ 40 mm${^2}$ with a 1 mm strip pitch. A planar Ge detector was placed behind the DSSDs to measure X rays and low-energy $\gamma$ rays. The planar Ge detector had an active area of 120 mm $\times$ 60 mm and thickness of 15 mm. A thin beryllium window separated the planar Ge detector from the GREAT vacuum chamber [27]. The data from all detectors were recorded by the triggerless Total Data Readout (TDR) system [28]. All events were time stamped with 10 ns precision. The data were analyzed using the GRAIN software package [29], the data analysis framework ROOT [30], and the fitting program HDTV [31].

**Fig. 1:**
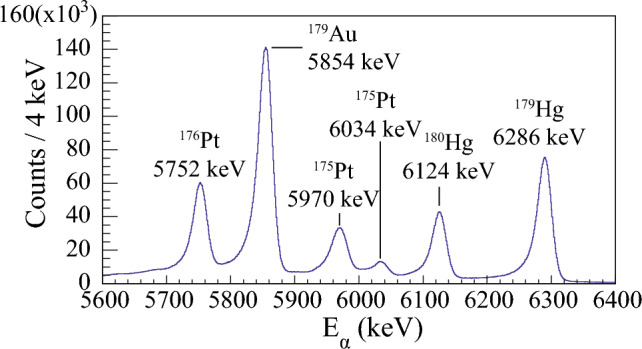
Energy spectrum of $\upalpha$ particles measured in the DSSD detector, vetoed by the MWPC events. The fusion-evaporation reaction $^{82}_{36}$Kr + $^{100}_{44}$Ru was used to produce nuclei of interest

## Experimental Results

**Fig. 2:**
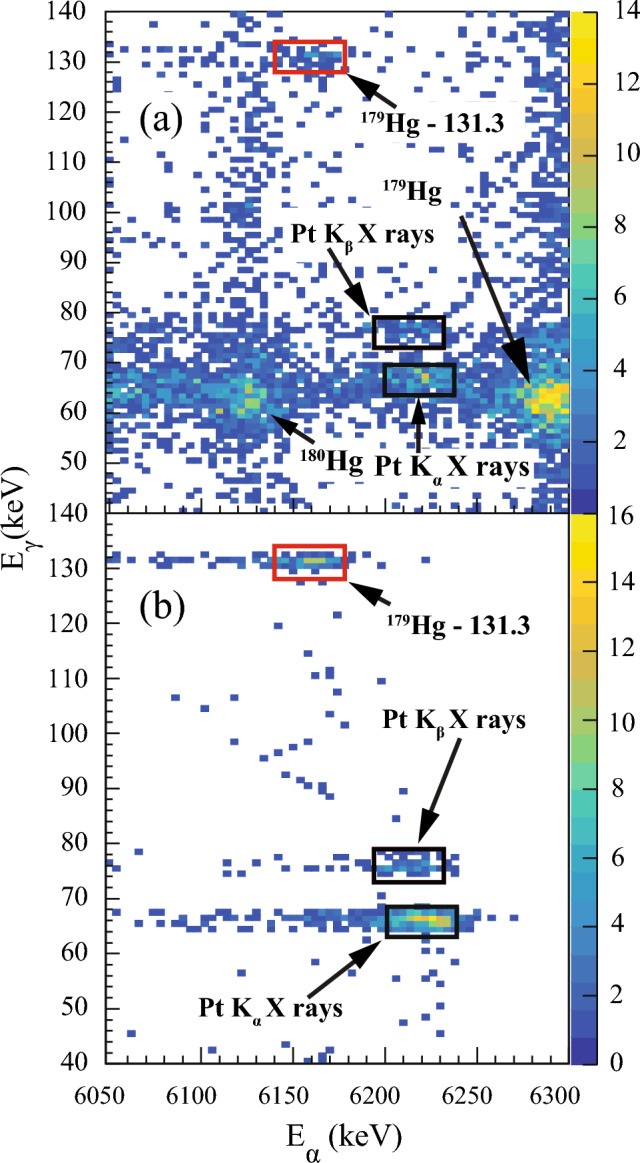
**a** The background subtracted $\upalpha$-$\gamma$ coincidence matrix measured with the DSSD and focal-plane planar Ge detector, and **b** the simulated data. The time conditions used to search for coincidences: $\varDelta t$($t_{\alpha }$ - $t_{ER}$ ) < 3 s and $\varDelta t$($t_\gamma$ - $t_\alpha$) < 200 ns. The red rectangle represents the newly observed $\upalpha$-$\gamma$ coincidence while black rectangles represent Pt K$_{\alpha }$ and Pt K$_{\beta }$ X rays. The data in panel **b** were simulated in GEANT4. The energy of the $\upalpha$ particles in coincidence with the Pt k$_\beta$ X rays is shifted due to the $\upalpha$-particle and conversion-electron energy summing. The experimental data were acquired during the $^{179}$Hg experiment

### $^{179}$Hg $\upalpha$ decay

Fig. 1 shows the energy spectrum of $\upalpha$ particles measured with the DSSD detector, vetoed by MWPC events. The peak at 6286(9) keV is associated with the g. s. $\rightarrow$ g. s. $\upalpha$ decay of $^{179}$Hg [12]. Note that only the 5960(10) keV and 6038(9) keV $\upalpha$ particles from the decays of $^{175}$Pt [11] were identified in the spectrum. The $\upalpha$ particles from the remaining two known $\upalpha$-decay branches of $^{175}$Pt cannot be clearly seen in the spectrum due to contamination of respective peaks by the $^{179}$Au activity. In order to search for the $\upalpha$-decay fine structure, data were sorted into a background-subtracted $\upalpha$-$\gamma$ coincidence matrix, with $\varDelta t$($t_{\alpha }$ - $t_{ER}$ ) < 3 s and $\varDelta t$($t_\gamma$ - $t_\alpha$) < 200 ns time conditions, see Fig. 2. Coincident events of 6156(10) keV $\upalpha$ particles and 131.3(5) keV $\gamma$ rays are clearly visible. In addition, characteristic Pt K$_\beta$ X rays are found to be in coincidence with the  6210 keV $\upalpha$ particles. They will be discussed in detail later in the text. The Q-value of the $\upalpha$ decay of $^{179}$Hg extracted from E$_\alpha$ = 6156 keV and E$_\gamma$ = 131.3 keV is 6428(10)keV. Within experimental uncertainties, this value corresponds to the *Q*-value of the known $^{179}$Hg $\upalpha$ decay, see Fig. 1, $Q_\alpha$(6286) = 6430(9) keV.

**Fig. 3:**
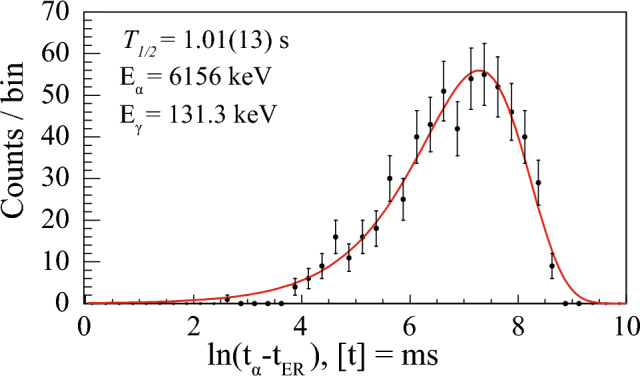
Time distribution of the $^{179}$Hg $\upalpha$ decay leading to the 131.3(5) keV excited state in $^{175}$Pt. A search time $\varDelta t$ = ($t_{\gamma }$ - $t_{\alpha _1}$) < 200 ns was used along with the red 2-dimensional gate used in Fig. 2

**Fig. 4:**
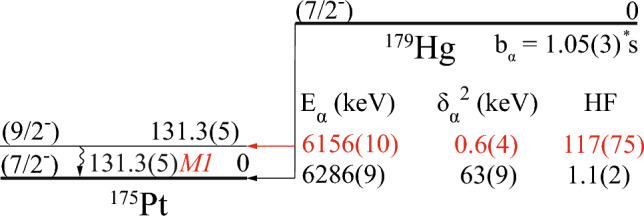
The $\upalpha$-decay scheme of $^{179}$Hg deduced in the present work. Ground-state properties of $^{179}$Hg and $^{175}$Pt are adopted from [13, 18], respectively. The values deduced or improved in the present work are highlighted in red

The observed coincidence between the 6156 keV $\upalpha$ particle, and the 131.3 keV $\gamma$-ray, are associated with the 131.3 keV excited state in $^{179}$Hg. The time distribution $\varDelta t$($t_\alpha$-$t_{ER}$) for the 6156 keV $\upalpha$ particles in coincidence with the 131.3 keV $\gamma$ rays is shown in Fig. 3. The data are plotted using a logarithmic time scale approach suitable for low-statistics data, as described in [32]. The fit gives a half-life of 1.01(13) s. Within experimental uncertainties, this agrees with the known half-life of the ground state of $^{179}$Hg, which is 1.05(3) s [15]. This strongly corroborates the previous interpretation of the 6156 keV $\upalpha$ particles. We assign the 6156 keV events to a new $\upalpha$-decay branch in $^{179}$Hg, thus making it the first observation of an $\upalpha$-decay fine structure in this nucleus. A revised decay scheme of $^{179}$Hg is constructed, see Fig. 4.

Characteristic Pt K X rays are observed in Fig. 2. These X rays occur due to atomic relaxation after internal conversion of the 131.3 keV transition. The $\upalpha$-electron summing effect, i.e., the addition of energies of conversion electrons to the energy of $\upalpha$ particles, causes shift of these $\upalpha$-particle energies towards higher energies in the $\upalpha$-$\gamma$ matrix, which is clearly documented with GEANT4 simulations [33], see Fig. 2. They can be used to deduce the K-internal conversion coefficient for the 131.3 keV transition. After correcting for detection efficiencies, simulated using a dedicated GEANT4 code, and accounting for the X-ray fluorescence yield [34], an experimental K internal conversion coefficient of $\alpha _{K, exp}$ (131.3 keV) = 3.9(23) was determined. This establishes an *M*1 character for the 131.3 keV transition, since theoretical values of $\alpha _{K, theo}$ for different transition types are: *E*1 = 0.1623(23), *E*2 = 0.468(7), *M*1 = 2.32(4) and $\alpha _{K, theo}$(*M*2) = 13.90(20), calculated with the BrIcc code [35]. Higher-order multipolarities are excluded due to the prompt nature of the 131.3 keV transition. We further validated this approach by simulating the $\upalpha$ decay of $^{177}$Pt with E$_\alpha$ = 5423(3) keV: 5/2$^-$ g.s. $\rightarrow$ 7/2$^-$ exc. state at 91.8(2) keV, which de-excites via emission of a 91.8(2) keV $\gamma$ ray down to the 5/2$^-$ g.s.. The experimental K conversion coefficient for this transition is $\alpha _K$ = 5.7(5), with an assigned $M1\,+\,E2$ multipolarity and a mixing ratio of 0.30(16) [36]. The conversion coefficient extracted from our simulation is $\alpha _K$ = 6.1(7), which is consistent with the previously measured value.

Finally, the deduced *M*1 multipolarity was used to calculate the branching ratio for the newly observed $\upalpha$-decay branch of $^{179}$Hg. A total $\upalpha$-decay branching ratio, $b_\alpha$ = 75(4) % [13] was assumed and divided as follows: (a) the number of $\upalpha$-particles populating the ground state in $^{175}$Pt, detected in the DSSD, see Fig. 1, and (b) the number of 131.3 keV $\gamma$ rays together with a number of conversion electrons obtained from Pt K$_\beta$ X rays, observed in Fig. 2, normalized to detection efficiency of the Planar Ge detector [37] and corrected for background contribution. This gives $b_\alpha$ = 0.20(8) % for the newly observed $\upalpha$ decay in $^{179}$Hg with an emitted $\upalpha$-particle energy of 6156 keV. Consequently, a branching ratio of the g. s. $\rightarrow$ g. s. $\upalpha$ decay of $^{179}$Hg with E$_\alpha$ = 6286 keV needs to be slightly reduced accordingly to 74.8(50) %. The deduced $\upalpha$-decay scheme along with the calculated decay parameters for the $^{179}$Hg $\upalpha$ decay are given in Table 1 and Fig. 4.

**Table 1:** Summary of the $^{179}$Hg $\upalpha$-decay characteristics deduced in the present work. Alpha-particle energy (E$_\alpha$), branching ratio ($b_\alpha$), reduced width ($\delta ^2$), and hindrance factor (HF), together with the energy level populated by the $\upalpha$ decay (E$_{level}$) and multipolarity ($M\lambda$) of the $\gamma$-ray transition de-exciting the energy level are shown

E$_{\alpha }$(keV)	$b_\alpha$ (%)	$\delta ^2$(keV)	HF	E$_{level}$ (keV)	Multipolarity
6286(9)	74.8(50)	63(9)	1.1(2)	0	
6156(10)	0.20(8)	0.6(4)	117(75)	131.3(5)	*M*1

### $^{177}$Au $\upalpha$ decay

**Fig. 5:**
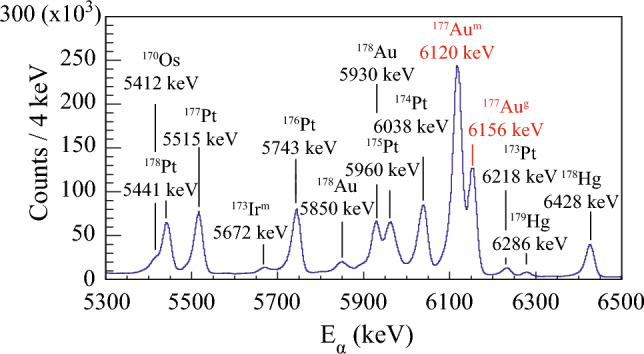
Energy spectrum of $\upalpha$ particles measured in the DSSD detector, vetoed by the MWPC events. The nuclei of interest were produced in the fusion-evaporation reaction $^{82}_{38}$Sr + $^{92}_{42}$Mo. Peaks belonging to known $\upalpha$ decays of $^{177}$Au are highlighted in red

**Fig. 6:**
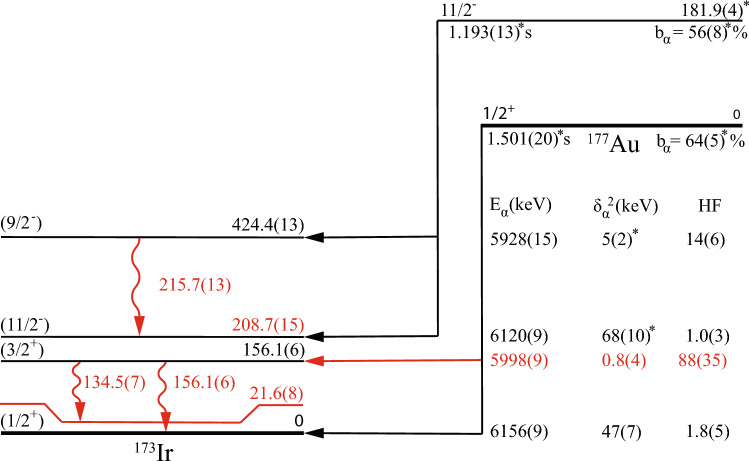
The $\upalpha$-decay scheme of $^{177}$Au deduced in the present work. Values marked by an asterisk are adopted from [23]. Values deduced or improved in the present work are highlighted with red color

**Fig. 7:**
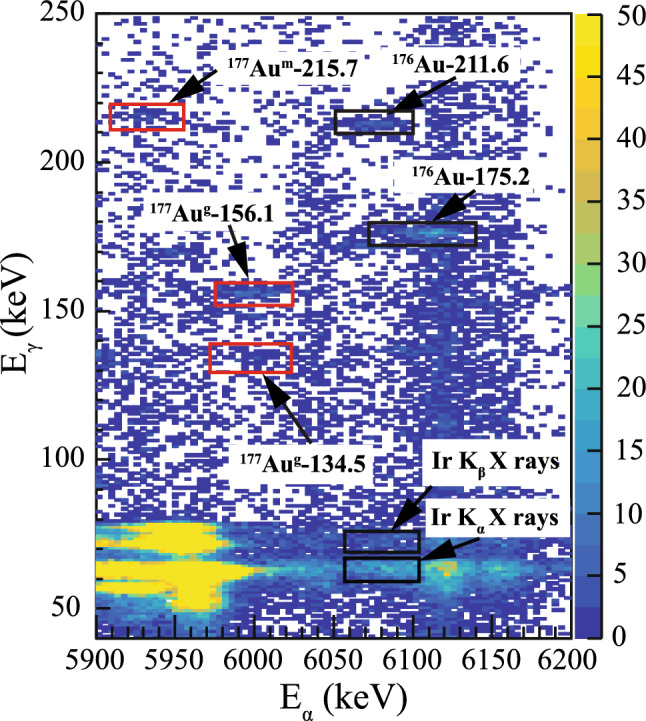
The background subtracted $\upalpha$-$\gamma$ coincidence matrix measured with the DSSD and focal-plane planar Ge detector. Time conditions used to search for coincidences: $\varDelta t$($t_{\alpha }$ - $t_{ER}$ ) < 4 s and $\varDelta t$($t_\gamma$ - $t_\alpha$) < 200 ns. The red rectangles represent new $\upalpha$-$\gamma$ coincidences relevant to the decay of $^{177}$Au, while the black rectangles highlight other strong coincidences along with the Ir K$_{\alpha }$ and Ir K$_{\beta }$ X rays. The coincidences shown in the matrix were produced in the fusion-evaporation reaction $^{82}_{38}$Sr + $^{92}_{42}$Mo

Figure 5 shows the spectrum of $\upalpha$-decay singles measured with the DSSD detector, vetoed by the MWPC. Dominant peaks are identified as known $^{177}$Au $\upalpha$ decays. The peak at 6120(9) keV is attributed to the $^{177}$Au$^m$ $\upalpha$ decay, as it matches well to the E$_\alpha$ = 6125(5) keV from the previous study [23]. The energy of E$_\alpha$ = 6156(9) keV is in a good agreement with the g. s. $\rightarrow$ g. s. $\upalpha$ decay of $^{177}$Au with the reported value of 6156(5) keV [23]. The $^{177}$Au $\upalpha$-decay scheme, constructed in the present work is presented in Fig. 6, and is discussed in detail in the following text. To search for $\upalpha$-decay fine structure, data were sorted into a background subtracted $\upalpha$-$\gamma$ coincidence matrix, with $\varDelta t$($t_{\alpha }$ - $t_{ER}$ ) < 4 s and $\varDelta t$($t_\gamma$ - $t_\alpha$) < 200 ns time conditions. The $\upalpha$-$\gamma$ matrix is shown in Fig. 7. In the matrix, three $\upalpha$-$\gamma$ coincidences marked by the red rectangles were attributed to the fine structure in the $\upalpha$ decay of $^{177}$Au. The matrix was produced from the events collected in the fusion-evaporations reactions $^{88}_{38}$Sr + $^{92}_{42}$Mo.

**Fig. 8:**
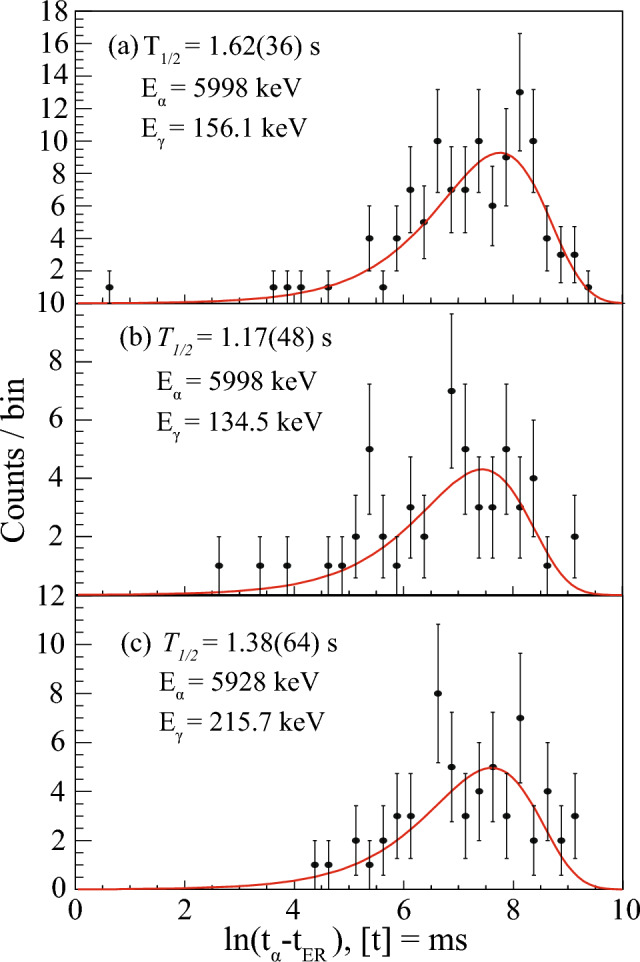
Time distributions of $^{177}$Au $\upalpha$ decays leading to the **a** 156.1(6) keV excited state with an emitted 156.1(6) keV $\gamma$ ray, **b** 156.1(6) keV excited state with an emitted 134.5(7) keV $\gamma$ ray and **c** 424.4(13) keV excited state with an emitted 215.7(13) keV $\gamma$ ray in $^{173}$Ir. Search times of $\varDelta t$ = ($t_{\gamma }$ - $t_{\alpha _1}$) < 200 ns were used along with the red 2-dimensional gates used in Fig. 7

The $\upalpha$-decay branch with E$_\alpha$ = 5928(15) keV $\upalpha$ decay was previously assigned as the decay of $^{177}$Au$^m$ feeding the 424.4(13) keV excited state in $^{173}$Ir [23]. Note that the excitation energy of the state has been determined in an in-beam study of $^{173}$Ir [38], and the decay from the $^{177}$Au isomer does not feed the g.s. in $^{173}$Ir. No $\gamma$-ray transition was previously seen to depopulate this excited state. In this work, the 5928 keV $\upalpha$ particles are seen in coincidence with the 215.7(13) keV $\gamma$ rays, see Fig. 7. This $\gamma$-ray energy matches well to the energy difference between two excited states with known energies of 424.4 keV and 213 keV [23]. The time distribution between the recoil implantation and subsequent $\upalpha$ decay was used to measure the half-life of the $\upalpha$-decay branch with E$_\alpha$ = 5928 keV in coincidence with the 215.7 keV $\gamma$ rays, see Fig. 7. The $\upalpha$-decay half-life was obtained from the fit to the data plotted according to the logarithmic time scale method [32], see Fig. 8, resulting in $\varDelta t$($t_\alpha$-$t_{ER}$) = 1.38(64) s. Within experimental uncertainties, this value corresponds to the known $T_{1/2}$(1.193 s) of $^{177}$Au$^{m}$. Therefore, a new 215.7 keV $\gamma$-ray transition was placed to depopulate the 424.4 keV excited state in $^{173}$Ir, feeding the 11/2$^-$ isomeric state, see Fig. 6. This establishes a more accurate excitation energy of 208.7(18) keV.

The $\upalpha$ decay with E$_\alpha$ = 5998(9) keV is seen in coincidence with the 156.1(6) keV $\gamma$ ray, see Fig. 7. Previously, the 6000(20) keV $\upalpha$ decay was suggested to feed the 155.4(10) keV excited state in $^{173}$Ir [23]. The value of $Q_{\alpha , tot}$ = $Q_{\alpha }$ (5998) + $E_\gamma$ (156) = 6293(9) keV is in a good agreement with $Q_\alpha$(6156) = 6299(9) keV. Additionally, the experimental half-life $T_{1/2}$ (5998 keV) = 1.62(36) s agrees with the known $T_{1/2}$(1.501 s) $\upalpha$ decay of $^{177}$Au$^{g}$, see Fig. 8. This confirms the tentatively assigned decay path in [23].

**Fig. 9:**
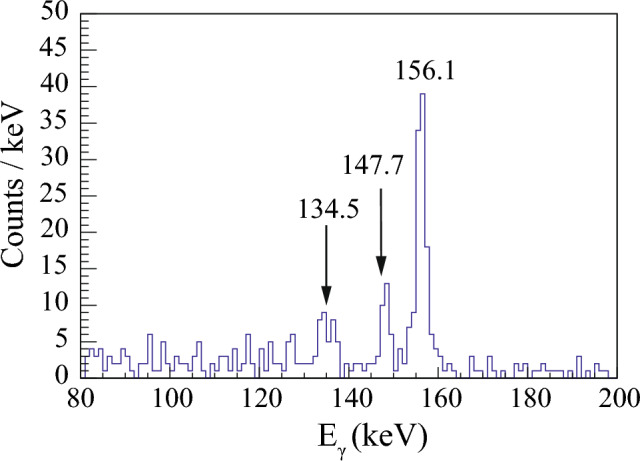
Energy spectrum of $\gamma$ rays in coincidence with the 5998 keV $\upalpha$ particles in the $\upalpha$-$\gamma$ coincidence matrix shown in Fig. 7. The presence of the 147.7 keV peak is explained as the 6006(9) keV $\upalpha$ particles of $^{181}$Hg populating the 147.4(10) keV excited state in $^{177}$Pt. The $^{181}$Hg was produced due to impurities in the target

The same $\upalpha$ decay with E$_\alpha$ = 5998 keV is seen in coincidence with a 134.5(7) keV $\gamma$ ray. A spectrum of $\gamma$ rays in coincidence with the 5998 keV $\upalpha$ particles is shown in Fig. 9. A $\gamma$ ray at 147.7(10) keV is attributed to the $\upalpha$-decay fine structure of $^{181}$Hg [11]. This isotope is present in our data due to target impurities. The $Q_{\alpha , tot}$ value is equal to $Q_{\alpha }$(5998) + $E_\gamma$ (134.5) = 6272(9) keV. The difference between this value and the value of $Q_\alpha$(6156) is 27(13) keV. The experimental half-life, gated on a $^{177}$Au$^g$ - 134.5 2-dimensional gate in Fig. 7, is $T_{1/2}$(5998) = 1.17(48) s, see Fig. 8. Therefore, the 134.5 keV transition is assigned to depopulate the 156.1 keV excited state down to a new 21.6(10) keV excited state in $^{173}$Ir.

Here, the method used to obtain the conversion coefficient in Sec. 3.1 gives $\alpha _K$ (156.1 keV) = 3.3(15). However, in this case, this method does not result in a precise value. The number of Ir K$_\beta$ X rays summed with the energy of $\upalpha$ particles is not derived only from the de-excitation of the 156.1 keV state down to the ground state. All of the aforementioned transitions contribute to the Ir K$_\beta$ X rays used to calculate the conversion coefficient mentioned above. It is therefore impossible to properly extract the conversion coefficient for each of these transitions separately. Therefore, the extracted conversion coefficient for the 156.1 keV transition is too large. Moreover, the 156.1 keV transition is seen as prompt; therefore, an *M*1+*E*2 character is tentatively assumed. Due to low statistics, the multipolarity of the 134.5 and 215.7 keV transitions could not be extracted from our data.

An identical approach to that used in Sec. 3.1 was used to obtain the branching ratios of the 5998 keV $\upalpha$ decay populating the 156.1 keV excited state in $^{173}$Ir. The number of 156.1 keV and 134.5 keV coincident events observed in Fig. 7 was used with assumed *M*1 multipolarity for both transitions. Under these assumptions, $b_\alpha$(5998) < 0.24%. The branching ratio of the 6156 keV $\upalpha$ decay populating the ground state in $^{173}$Ir had to be reduced to 63.8(41)%. Results of the $^{177}$Au decay analysis are summarized in Fig. 6 and Table 2.

**Table 2:** Summary of the $^{177m,g}$Au $\upalpha$-decay characteristics deduced in this work. Alpha-decay energy (E$_\alpha$), branching ratio ($b_\alpha$), reduced width ($\delta ^2$), and hindrance factor (HF), together with the excited state populated by the $\upalpha$ decay (E$_{level}$) and energy of $\gamma$ ray (E$_{\gamma }$) emitted from the populated state are listed. Values marked by an asterisk are taken from [23], while the other values are from this work

E$_{\alpha }$(keV)	$b_\alpha$ (%)	$\delta ^2$(keV)	HF	E$_{level}$ (keV)	E$_{\gamma }$ (keV)
6156(9)	63.8(41)	47(7)	1.8(5)	0	0
5998(9)	< 0.24	0.8(4)	88(35)	156.1(6)	156.1(6)
					134.5(7)
6120(9)	55.3(79)	68(10)*	1.0(3)	208.7(15)	
5928(15)	0.7(3)	5(2)*	14(6)	424.4(13)*	215.7(13)

## Discussion

### A. $^{179}$Hg $\upalpha$ decay

The decay scheme of $^{179}$Hg established in this work is shown in Fig. 4. The reduced $\upalpha$ decay widths $\delta ^2$ for both $\upalpha$ decays of $^{179}$Hg were calculated using the approach prescribed by Rasmussen [7]. In these calculations, a change in angular momentum of $\varDelta L$ = 0 was assumed between the parent and child nuclei. The hindrance factors were determined by comparing the reduced $\upalpha$-decay widths to those of unhindered 0$^+$ to 0$^+$ decays in the neighbouring even-even nuclei, $^{178}$Hg and $^{180}$Hg. Reduced $\upalpha$ decay widths of $\delta ^2$ = 0.6(4) keV and 63(9) keV were calculated for E$_\alpha$ = 6156 and 6286 keV, respectively. This gives hindrance factors of HF = 117(75) and 1.1(2) for the respective decays. The calculated hindrance factor corroborates the 9/2$^-$ spin-parity assignment for the 131.3 keV excited state in $^{175}$Pt, as suggested in [18].

Transitions with properties similar to the one discussed above were also observed in $^{177}$Pt isotones. In $^{173}$Os, a de-excitation of the 219.6 keV $9/2^-$ excited state down to the $7/2^-$ excited state at an energy of 91.6 keV by emission of a 128 keV $\gamma$ ray was reported [39]. This 128 keV transition is an interband transition in a collective band (band 2 in Fig. 3 of [39]) in $^{173}$Os. The $^{175}$Pt nucleus has a 0.7% probability to decay via $\upalpha$ decay. Unfortunately, only $\upalpha$ decays populating the $5/2^-$ ground state dominated by the 5/2$^-$ [523] Nilsson configuration and the $7/2^-$ excited state at 92 keV in $^{173}$Os have been observed so far [11]. Similarly, a 131.4 keV transition depopulating the $9/2^-$ excited state at 233.2 keV down to the $7/2^-$ excited state at 101.9 keV in $^{171}$W was reported in [40]. The 131.4 keV transition in $^{171}$W connects two negative-parity rotational bands denoted as E and F, see Fig. 1 in [40], built on top of the $5/2^-$ ground state, dominated by the 5/2$^-$ [523] Nilsson configuration. The $^{175}$Os nucleus decays solely via $\beta ^+/EC$ decay, therefore no comparison with the $\upalpha$ decay discussed here could be made. However, the $9/2^-$ excited states are populated by a $\beta ^+/EC$ decay in both cases. Remarkably, in both cases, the two transitions mentioned above are part of the ground state rotational bands and were assigned an *M*1 character [39, 40].

### B. $^{177}$Au $\upalpha$ decay

An identical approach as for the $^{179}$Hg $\upalpha$ decay was used to calculate the $^{177}$Au $\upalpha$-decay characteristics. For the observed $\upalpha$ decays of $^{177}$Au, we calculated the following parameters: E$_{\alpha }$ = 5998 keV, the reduced width $\delta ^2$ = 0.8(4) keV and hindrance factor HF =88 (35); E$_{\alpha }$ = 6156 keV, the reduced width $\delta ^2$ = 47(7) keV and hindrance factor HF = 1.8(5). The unhindered E$_\alpha$ = 6156 keV $\upalpha$ decay indicates a large overlap in the initial and final state wave functions. On the other hand, the hindered E$_\alpha$ = 5998 keV $\upalpha$ decay suggests different spins of the initial and final states. Regarding the $^{177}$Au$^m$ $\upalpha$ decays, hindrance factors were calculated using values of I$_{\alpha , rel}$ and $\delta ^2$ = 68(10) keV adopted from [23]. For the E$_\alpha$ = 6120 keV $\upalpha$ decay populating the 208.7 keV excited state in $^{173}$Ir, HF = 1.0(3) was calculated. This suggests a large overlap of the initial and final state wave functions. A value of HF = 14(6) was calculated for the $\upalpha$ decay with E$_\alpha$ = 5928 keV populating the 424.4 keV excited state in $^{173}$Ir. Such HF value suggests a spin change in the $\upalpha$ decay process.

Regarding the 21.6 keV excited state, in our data the *Q*-value analysis along with the measured half-life agree with a 134.5 keV $\gamma$ ray emitted in a transition connecting the 156.1 keV and 21.6 keV excited states. On the other hand, due to the summing effect of the conversion and Auger electrons emitted in the 21.6 kev transition, the energy of alpha particles in coincidence with the 134.5 keV $\gamma$ rays should be increased by $\sim$ 20 keV. A possible explanation is that if the 21.6 keV excited state is isomeric, the summing effect would not be observed in the present experiment. For this reason, the 134.5 keV transition was assigned to depopulate the 156.1 keV excited state down to the 21.6 keV excited state. Based on analogy with neighbouring isotopes, the state is assigned as the 3/2$^+$ member of the ground state configuration. A similar transition was observed in neighbouring nuclei. First, in the parent nucleus $^{177}$Au, an excited state at 25.7 keV was identified [41]. The difference is that this excited state was seen to be fed by the 265.4 keV transition depopulating the 5/2$^+$ excited state at 290.2 keV. The 25.7 keV transition was not observed in the in-beam $\gamma$-ray study [41]. The existence of the 25.7 keV state was identified by the energy difference of parallel 264.5 keV and 290.2 keV transitions and their coincidence with a 452.6 keV transition feeding the 5/2$^+$ state. In $^{175}$Ir, a 3/2$^+$ excited state at the energy of 26.1 keV was observed [23]. The position and de-excitation of the 26.1 keV excited state was first observed in [42], where a cascade of 45.2 keV and 26.1 keV transitions was seen to depopulate a 71.3 keV excited state. An *M*1 character was assigned to the 26.1 keV transition. In $^{179}$Au, the 27.1 keV excited state was reported [43]. The corresponding 27.1 keV *M*1 transition was identified as well. Remarkably, in all three cases an $\approx$ 26 keV transition depopulates a 3/2$^+$ excited state down to a 1/2$^+$ ground state. Therefore, the neighbouring nuclei strongly support the existence of a similar excited state at 21.6 keV in $^{173}$Ir.

The newly observed 215.7 keV transition connects the 9/2$^-$ band head of the 1$h_{9/2}$ band with the 11/2$^-$ state of the 1$h_{11/2}$ structure. While the 1$h_{9/2}$ structure has an intruder and thus particle character, the 1$h_{11/2}$ represents a proton-hole configuration. Transitions connecting such structures are known to be hindered. E.g., in $^{189}$Au, the 9/2$^-$ $\rightarrow$ 11/2$^-$ *M*1 + *E*2 transition with B(*M*1) = 4.8(17)$\times$10$^{-5}$ W.u. and B(*E*2) = 0.3$^{+0.4}_{-0.3}$ W.u. This results in an isomeric character of the 9/2$^-$ state in $^{189}$Au with half-life of 190(15) ns. If we apply these values to the present case of $^{173}$Ir, a lifetime of approximately 20 ns would be expected. This would be sufficient to cause the non-observation of this transition in an in-beam study, but it is still possible to observe it in the decay study. Note that it was not possible to determine the lifetime of the 9/2$^{-}$ state from the present data because of insufficient time resolution of our system.

## Summary

The $\upalpha$-decay fine structure of $^{179}$Hg and $^{177}$Au was studied by means of decay spectroscopy. In particular, ER-$\alpha _1$-$\alpha _2$ correlations and $\upalpha$-$\gamma$ coincidences were investigated. For $^{179}$Hg a new $\upalpha$-decay branch with E$_\alpha$ = 6156(10) keV was identified, feeding a (9/2$^-$) excited state at an excitation energy of 131.3(5) keV in $^{175}$Pt. The partial branching ratio of the new $\upalpha$-decay branch was determined to be 0.20(8) %. The reduced width and hindrance factor were calculated to be 1.1(7) keV, and 117(75), respectively. The HF = 117(75) indicates a spin change between the $^{179}$Hg ground state and the 131.3(5) keV excited state in $^{175}$Pt. An internal conversion coefficient $\alpha _K$ = 3.9(23) for the 131.3(5) keV transition in $^{175}$Pt was deduced for the first time. It confirms an *M*1 multipolarity assignment for this transition, corroborating the previous assignment based on an angular distribution measurement.

In the $\upalpha$ decay of $^{177}$Au, a new $\upalpha$-decay branch with E$_\alpha$ = 5998(9) keV was observed. The characteristics of this $\upalpha$ decay were calculated as follows: b$_\alpha$ < 0.24 %, $\delta ^2$ = 0.8(4) keV and HF = 88(35). This $\upalpha$ decay populates a 156.1(6) keV excited state in $^{173}$Ir. Two possible de-excitation paths were observed to depopulate the 156.1(6) keV excited state. First, a 156.1(6) keV transition feeding the ground state in $^{173}$Ir was identified. The second de-excitation path via a 134.5(7) keV transition feeding a 21.6(10) keV excited state was assigned, on the basis of similarities with neighbouring nuclei. Moreover, in this work, the populated 21.6(10) keV excited state is presumed to be isomeric due to the non-observation of the conversion electron summing effect.

Finally, a 215.7(13) keV $\gamma$-ray transition de-exciting the (9/2$^-$) intruder state and feeding the (11/2$^-$) state in $^{173}$Ir was observed for the first time. Comparison between in-beam experiment and the present data indicate an isomeric character of the 9/2$^-$ state.

## Data Availability

Data will be made available on reasonable request. [Author’s comment: The datasets generated during and/or analysed during the current study are available from the corresponding author on reasonable request.]

## Code Availability Statement

This manuscript has no associated code/software. [Author’s comment: Code/Software sharing not applicable to this article as no code/software was generated or analysed during the current study.]
